# Sustained Aeration of Infant Lungs (SAIL) trial: study protocol for a randomized controlled trial

**DOI:** 10.1186/s13063-015-0601-9

**Published:** 2015-03-15

**Authors:** Elizabeth E Foglia, Louise S Owen, Marta Thio, Sarah J Ratcliffe, Gianluca Lista, Arjan te Pas, Helmut Hummler, Vinay Nadkarni, Anne Ades, Michael Posencheg, Martin Keszler, Peter Davis, Haresh Kirpalani

**Affiliations:** Division of Neonatology, The Children’s Hospital of Philadelphia, 34th and Civic Center Blvd., 2nd Floor Main Building, Philadelphia, PA 19104 USA; Department of Pediatrics, University of Pennsylvania Perelman School of Medicine, 34th and Civic Center Blvd, Philadelphia, PA 19104 USA; Department of Newborn Research, Royal Women’s Hospital, 20 Flemington Road, Parkville, VIC 3052 Australia; University of Melbourne, Grattan Street, Parkville, VIC 3010 Australia; Murdoch Children’s Research Institute, Royal Children’s Hospital, 50 Flemington Road, 9th Floor, Parkville, VIC 3052 Australia; Department of Biostatistics and Epidemiology, University of Pennsylvania Perelman School of Medicine, Blockley Hall, 423 Guardian Dr., Philadelphia, PA 19104 USA; Division of Neonatology, ‘VBuzzi’ Children’s Hospital, Via Castelvetro 32, 20154 Milan, Italy; Division of Neonatology, Department of Pediatrics, Leiden University Medical Center, Albinusdreef 2, 2333 ZA Leiden, Netherlands; Division of Neonatology and Pediatric Critical Care, Department of Pediatrics, Children’s Hospital University of Ulm, Eythstrasse 24, Ulm, 89081 Germany; Department of Anesthesiology and Critical Care Medicine, The Children’s Hospital of Philadelphia, 34th and Civic Center Blvd, 8th Floor Main Building, Philadelphia, PA 19104 USA; Alpert Medical School of Brown University, 222 Richmond St, Providence, RI 02903 USA; Division of Neonatology, Women and Infants Hospital of Rhode Island, 101 Dudley Street, Providence, RI 02905 USA

**Keywords:** Preterm infants, Resuscitation, Bronchopulmonary dysplasia, Sustained inflation, Continuous positive airway pressure

## Abstract

**Background:**

Extremely preterm infants require assistance recruiting the lung to establish a functional residual capacity after birth. Sustained inflation (SI) combined with positive end expiratory pressure (PEEP) may be a superior method of aerating the lung compared with intermittent positive pressure ventilation (IPPV) with PEEP in extremely preterm infants. The Sustained Aeration of Infant Lungs (SAIL) trial was designed to study this question.

**Methods/Design:**

This multisite prospective randomized controlled unblinded trial will recruit 600 infants of 23 to 26 weeks gestational age who require respiratory support at birth. Infants in both arms will be treated with PEEP 5 to 7 cm H_2_O throughout the resuscitation. The study intervention consists of performing an initial SI (20 cm H_2_0 for 15 seconds) followed by a second SI (25 cm H_2_O for 15 seconds), and then PEEP with or without IPPV, as needed. The control group will be treated with initial IPPV with PEEP. The primary outcome is the combined endpoint of bronchopulmonary dysplasia or death at 36 weeks post-menstrual age.

**Trial Registration:**

www.clinicaltrials.gov, Trial identifier NCT02139800, Registered 13 May 2014

**Electronic supplementary material:**

The online version of this article (doi:10.1186/s13063-015-0601-9) contains supplementary material, which is available to authorized users.

## Background

At birth, the newborn infant faces immediate and significant challenges for successful transition to the extrauterine environment. The critical physiological tasks to accomplish are to aerate the liquid-filled lung and thereby maintain aerated lung volume to establish a functional residual capacity (FRC). While term infants begin to establish the FRC with the first breath after birth [1], preterm infants are hampered by a greater instability of the thorax [2-4], limited muscle strength, and immature epithelial sodium channels, surfactant composition and production [5]. Use of positive end expiratory pressure (PEEP) during intermittent positive pressure ventilation (IPPV) or use of continuous positive airway pressure (CPAP) alone is currently recommended after birth to facilitate alveolar recruitment and to avoid baro-volu-trauma from mechanical ventilation [6]. However, well-performed trials indicate that despite a strategy of CPAP use after birth in extremely low gestational age neonates, rates of bronchopulmonary dysplasia (BPD) or death at 36 weeks postmenstrual age (PMA) remain high, ranging from 41 to 64% [7-9].

An additional approach to promote lung liquid clearance and aeration, ‘sustained inflation’ (SI), holds an inflating pressure for a period in order to facilitate lung fluid clearance and to establish the FRC. Initial human studies described inflations of up to 5 seconds in term infants [10]. Subsequently, SI has been increased to up to 30 seconds in animal models [11-16]. Earlier studies comparing SI to conventional resuscitative measures in preterm infants have shown promise but have been hampered by major limitations, including observational study design, lack of PEEP use in the control groups, early stopping, or lack of power to detect the outcomes of BPD or death at 36 weeks PMA [17-20].

To date, no trial has been powered to directly compare initial SI plus PEEP to initial IPPV plus PEEP for the important composite outcome of BPD and death. We designed the Sustained Aeration of Infant Lungs (SAIL) study to determine whether initial SI with PEEP is superior to initial IPPV with PEEP to prevent BPD or death in extremely preterm infants.

### Aims

The primary objective of this study is to compare the rate of BPD or death at 36 weeks PMA in infants born at 23^0/7^ to 26^6/7^ weeks gestational age (GA) with inadequate respiratory effort immediately after birth who receive either initial SI with PEEP or initial IPPV with PEEP as the lung recruitment strategy.

## Methods/design

This is a large, international, multicenter, prospective, unblinded, randomized controlled trial in extremely preterm infants at birth.

### Population

Patients will be recruited in this multisite international trial in 13 tertiary level delivery hospitals located in USA, Canada, Australia, the Netherlands, Italy and Germany.

### Inclusion criteria

Inclusion criteria are as follow:

1. GA at least 23 weeks but less than 27 completed weeks by best obstetrical estimate.
2. Requiring resuscitation/respiratory intervention at birth due to inadequate respiratory effort (defined as apnea or gasping) or heart rate (HR) <100 beats per minute (bpm).

### Exclusion criteria

Exclusion criteria are as follow:

1. Resuscitative care not provided, based on attending neonatologist or family’s decision.
2. Refusal of informed consent.
3. Known major congenital anomalies or pulmonary hypoplasia.
4. Infants born to mothers who are unable to give informed consent for their medical care and who do not have a surrogate guardian.

### Recruitment

Informed consent will be obtained using one of two methods. In some sites, all consents will be obtained in the antenatal period, whereby a member of the study team will approach parents of potentially eligible infants with threatened preterm delivery between 23^0/7^ and 26^6/7^ GA to offer study participation and to obtain written informed consent. At some sites Ethics Boards have indicated that waiver of prospective consent (or deferred consent), may be obtained, whereby eligible infants will be randomized to the SI intervention or standard care immediately after birth. As soon as possible following the resuscitation and study intervention, a member of the study team will approach the infant’s parents to obtain informed consent for continued study participation and data collection.

Twelve clinical sites and the data coordinating center have Ethics Board approval at the time of this submission (Additional file 1: Table S1); all remaining sites will obtain Ethics Board approval prior to initiating subject recruitment.

### Randomization

A permuted block randomization will be employed with unequal blocks of varying sizes, and an allocation ratio within each block of 1. Pre-randomization stratification is by gestational age (23^0/7^ to 24^6/7^ and 25^0/7^ to 26^6/7^) and by study site. Since time does not permit computer randomization, sealed opaque allocation envelopes will be color-coded (by strata) and kept in a standard location in close proximity to the resuscitation suite. Randomization will occur after birth once the final assessment of eligibility has been made.

### Blinding

The study intervention is unblinded. However, to protect against potential bias in outcome ascertainment, the primary outcome is either objective (death) or performed blinded to the initial resuscitation arm (that is, Oxygen Reduction Test [21] at 36 weeks PMA).

### Intervention

After birth and cord clamping as per each unit’s normal practice, potentially eligible infants (23^0/7^ to 26^6/7^ weeks GA) will be taken to a resuscitation trolley, placed in a plastic wrap, stimulated, and have a pulse oximeter probe placed on the right hand. After ensuring airway patency, the infant will be placed on local standard interface (facemask, nasopharyngeal tube, or nasal prong) with CPAP at 5 to 7 cm H_2_O and FiO_2_ 0.3 via a T-piece resuscitator. The resuscitation team will assess the respiratory effort and heart rate.

**Figure 1:**
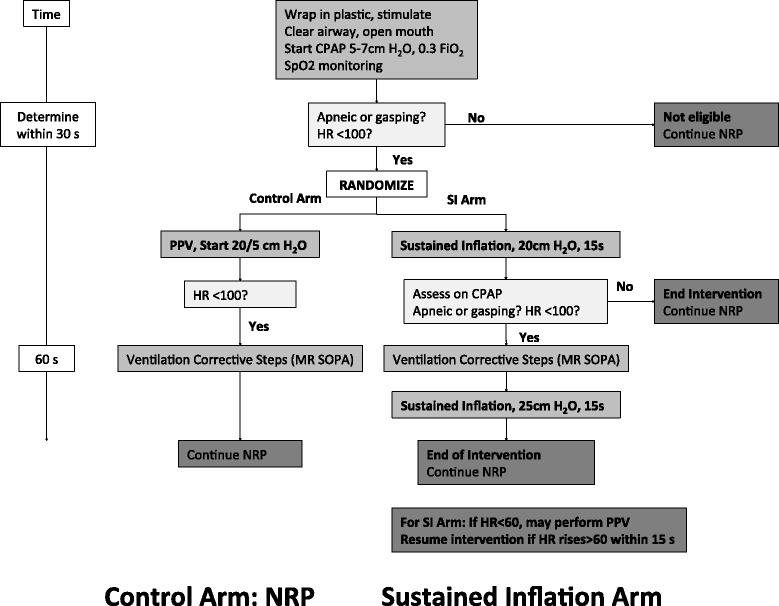
**Resuscitation algorithm.** abbreviations in figure: CPAP, continuous positive airway pressure; FiO_2_,fraction of inspired oxygen; HR, heart rate; NRP, neonatal resuscitation program; PPV, positive pressure ventilation; SI, sustained inflation; SpO_2_, oxygen saturation.

Infants with adequate respiratory effort and HR >100 bpm will not meet inclusion criteria and will not be enrolled in the trial. Infants with inadequate respiratory effort (defined as apnea or gasping respirations) or HR <100 bpm will be eligible for trial enrollment. At the moment each infant is deemed eligible for enrollment (within 30 seconds of arrival on the resuscitation trolley), the randomization envelope will be opened and the treatment allocation will be announced. Infants randomized to the control arm will be treated with IPPV with PEEP according to the neonatal resuscitation program (NRP) or equivalent local recommendations [22]. Infants in the SI arm will be treated with an initial SI of 20 cm H_2_O for 15 seconds followed by assessment on CPAP. For infants who continue to have inadequate respiratory effort and/or bradycardia, ventilation corrective steps will be performed as needed and a second SI of 25 cm H_2_O for 15 seconds will be performed (Figure 1). At that point, the intervention is complete, and all subsequent care will follow local resuscitation protocols.

### Extubation guidelines

Because the duration of invasive respiratory support is a critical end point, guidelines related to extubation are defined. Extubation should be attempted within 24 hours after meeting all the following criteria: PCO_2_ ≤ 55 mm Hg and a pH ≥7.25, FiO_2_ of ≤0.4 with SpO_2_ of ≥88%, mean airway pressure of ≤8 cm of water, and hemodynamic stability. All infants will have received caffeine prior to an extubation attempt. Similar considerations prompted us to provide consensus guidelines for intubation, including: FiO_2_ ≥ 0.5 to maintain SpO2 ≥ 88%, pH ≤7.22, PCO_2_ ≥ 70 mm Hg, >1 apneic event requiring IPPV within 6 hours, ≥6 apneic events requiring stimulation within 6 hours, cardiovascular instability, or need for surgery.

### Primary outcome measure

The primary outcome is the composite outcome of either BPD or death, as assessed by standard oxygen reduction test at 36 weeks PMA [21].

### Secondary outcomes

We will capture important secondary outcomes, including short-term respiratory morbidity and potential harms from delivery room (DR) interventions (Additional file 2: Table S2).

### Sample size

Using data from other recent large neonatal randomized trials with similar populations, for example, the NICHD SUPPORT trial [7] and the COIN trial [8], we estimate that the baseline rate of BPD/death at 36 weeks PMA in the control arm is 65%. In order to have 80% power to detect an absolute risk reduction of 12.5% (or 20% reduction in the baseline rate) with two planned interim analyses, 263 subjects per treatment arm are required. Multiples will be randomized as a set to the same study arm, requiring an inflation of the estimate by 1.12 to allow for the design effect due to clustering [23-25]. Thus, the calculated sample size is 296 infants per arm, rounded to a final recruitment target of 300 infants per arm.

### Data collection

With the exception of the data related to the screening log and pertaining to the initial SI and IPPV maneuvers, all remaining data will be obtained from the clinical records (Additional file 3: Table S3).

### Statistical analysis

Analyses will be performed on Statistical Analysis Software (SAS) using an ‘as randomized’ (formerly called ‘intention-to-treat’) principle to compare the primary outcome between treatment arms. Logistic regression will be used to control for covariates and identify potential effect modification. Potential covariates include sex, gestational age, initial heart rate, maternal corticosteroid use, and small for gestational age. The logistic regression will be estimated using a generalized estimating equation in order to adjust for the inherent correlation expected with multiples [23]. Regression diagnostics will assess model adequacy and potential outlying or influential data points. If there are clinical or demographic characteristic imbalances between the two treatment arms, a propensity score analysis will be used to assess the sensitivity of the results to any treatment allocation biases.

Secondary outcomes will be analyzed using similar procedures to the primary outcome. Comparisons between treatment arms will use logistic regression (dichotomous outcomes), linear regression (continuous outcomes), or survival analysis (survival time outcomes), as appropriate.

### Safety

A data and safety monitoring committee (DSMC) will protect all study subjects and monitor the overall conduct of the trial. The DSMC will review all adverse events within the first 10 days post-delivery room intervention. This and pre-determined early stopping rules for trial cessation will ensure safety [26]. Adverse events and their relationship to study, severity, time of experience, expectation, actions taken to resolve the event and final outcome will be recorded as documented in the medical record. All Serious Adverse Events (SAE) will be sent within 72 hours to the DSMC.

### Interim analysis

Two interim statistical analyses will be performed and will be reviewed by the DSMC after approximately 1/3 and 2/3 of the total subjects have completed their primary outcome. The primary outcome for the interim analyses will be the comparison of BPD/death between the treatment arms using a generalized estimating equation (GEE) model for BPD/death versus treatment. An approximate O’Brien-Fleming boundary will be used to calculate the nominal significance level to which interim *P* values are compared (Additional file 4: Table S4) [27]. To further ensure frequent safety monitoring, rates of any air-leak (pneumothorax, pulmonary interstitial emphysema [PIE]), and/or other serious adverse events will be assessed after 100 subjects have completed the primary outcome.

### Duration of study

The projected study duration is 5 years, including 2½ years of subject recruitment.

### Quality control and quality assurance procedures

Comprehensive education and training was undertaken to ensure technical proficiency and protocol compliance at all sites. Experienced study members ran a ‘boot-camp’ at the start of the study for site primary investigators (PIs), who are gold standard trainers at their local sites. Further, SI simulation curriculum and a training video were created for initial and refresher training for clinical personnel. In addition, a limited run-in period during which each site PI is present for all study interventions for the first 5 to 10 subjects per site was implemented in order to establish a level of comfort and to overcome the learning curve effect. Last, at sites with video recording capability during delivery room resuscitation, a single intervention picked randomly per 2 months will be videotaped and reviewed for reproducibility and consistency.

### Data processing, monitoring, and security

Data will be primarily managed using REDCap (Research Electronic Data Capture) [28] electronic data capture tools hosted by the University of Pennsylvania. REDCap is a secure, web-based application designed to support data capture for research studies and provides the following: 1) an intuitive interface for validated data entry; 2) audit trails for tracking data manipulation and export procedures; 3) automated export procedures for seamless data downloads to common statistical packages; and 4) procedures for important data from external sources. A data monitoring plan will serve as a reference guide for the development of case report forms, data handling conventions, reporting, data dictionaries, and supporting meta data. Access to direct identifiers will be limited to staff who meet all relevant training requirements and who must have access to these identifiers for quality control and monitoring. All data with identifiers will be stored on firewall-protected secure servers.

## Discussion

### Methodological considerations

The design and implementation of this trial present unique methodological considerations. We discuss three major challenges encountered during the design of the SAIL study.

#### Defining SI

The optimal peak inflation pressure (PIP) and duration of inflation required to aerate, but not overdistend, the immature lung are unknown. Models of SI in preterm animals report using inflation pressures as high as 35 to 40 cm H_2_O to aerate the lung after birth [12,13,16]. However, published studies of SI in preterm infants used lower inflation pressures, ranging from 20 cm H_2_O to 25 cm H_2_O for the first inflation, and escalating to pressures ranging from 25 to 30 cm H_2_O, with duration of SI ranging from 10 to 15 seconds [17-19].

Experience from clinical sites where SI is routinely used suggests that increased rates of air leaks (pneunomothorax and pulmonary interstitial emphysema) are associated with inflation pressures of 30 cm H_2_O (Personal communication, Hummler H). By consensus, we chose to adopt a progressive and cautious approach to the SI maneuver, starting with an initial SI inflation pressure of 20 cm H_2_O for 15 seconds, followed by a second SI inflation pressure of 25 cm H_2_O for 15 seconds in infants without an adequate clinical response to the first SI. We also excluded infants with adequate respiratory effort who have already partially aerated their lungs and thus may have better lung compliance, making them more susceptible to over-distention and air leaks. We have also included a comprehensive data safety monitoring plan for this study to closely monitor for evidence of increased air leaks and other adverse events proximal to the delivery room intervention.

#### Developing the SI intervention algorithm

The SI intervention algorithm needed to balance several objectives. We adhered to current International Liaison Committee on Resuscitation (ILCOR) consensus on science treatment recommendations and NRP guidelines for ethical reasons and to maintain equipoise. It was important that the algorithm be as clear as possible to facilitate training and clinician compliance with the protocol and be feasible within the existing framework of delivery room resuscitation at all the study sites.

Since this is an international multisite trial, we encountered variability in the delivery room resuscitation practices across SAIL study sites. While all sites’ practices are grounded in the ILCOR treatment recommendations, current variations include the following: resuscitation team composition; interface used for non-invasive ventilation (facemask versus nasal prong or nasopharyngeal tube); mode of delivering SI or IPPV (T-piece resuscitator versus ventilator); peak pressures and inflation times used during IPPV; and current utilization of IPPV or SI as the standard initial approach during DR resuscitation of extremely preterm infants. Of those sites where SI is currently used in practice, some sites utilize SI exclusively to aerate the lungs, and other sites interpose SI with IPPV during DR resuscitation.

Development of the intervention algorithm therefore required iterative discussions to build consensus among trial participants. Of particular concern was the question of how to approach the infant who remains apneic and bradycardic after the first SI. Proposed options at that point of the algorithm included proceeding directly to a second SI with a higher inflation pressure versus performing IPPV. If the SAIL study hypothesis is correct, SI is the preferred modality to aerate the preterm lung, and such an infant would benefit from an additional SI at a higher PIP to fully aerate the lung. Additionally, performing IPPV within the SI algorithm might dilute the treatment effect of SI. Last, clinical assessment of HR alone is imprecise and often underestimates true HR, [29], and HR data from pulse oximetry monitoring may not be available to clinicians at that point in the algorithm [30]. Proceeding directly to the second SI avoids potentially unnecessary deviations in the protocol based on inaccurate clinical assessments of HR.

However, countering these arguments was the understanding that it would be difficult for a resuscitation team accustomed to performing IPPV in apneic infants to withhold IPPV from a profoundly bradycardic and apneic newborn. There were also concerns of whether performing a second SI would delay ventilation and improvement in HR. Finally, an additional advantage of allowing IPPV is that it provides an opportunity to assess the quality of noninvasive ventilatory support (that is, chest rise, facemask leak, and airway obstruction) and therefore offers an opportunity for clinicians to correct these impediments before initiating a second SI.

Therefore, we decided to allow the following safety caveat in the SI algorithm to address these concerns: for infants who continue to have inadequate respiratory effort or who remain bradycardic (HR <100 bpm) after the first SI, corrective ventilation steps should be performed to ensure airway patency and adequate mask seal followed by a second SI with a higher PIP of 25 cm H_2_O. In addition, IPPV *may be performed at any point* if the infant’s HR is <60 bpm. If the HR recovers within 15 seconds of initiating IPPV, providers will resume the SI algorithm. If the HR does not rise after 15 seconds of IPPV, the intervention is complete and providers will continue the NRP recommended practices. Data monitoring will include regular audits of interventions performed during the SI algorithm to ensure that frequent deviations from the protocol are not occurring. If sites are identified as having a disproportionate number of infants randomized to the SI arm who receive IPPV, targeted feedback and educational interventions for that site will be performed to correct this trend.

#### Defining inadequate respiratory effort

Determining what constitutes inadequate respiratory effort, and thus eligibility for trial enrollment, presents a challenge. The target study population includes those infants with inadequate lung aeration after birth. This group encompasses infants who are persistently apneic after birth but also includes infants with ineffective respiration defined as gasping, irregular respirations, or very labored breathing. This last group, comprising infants with persistent but very labored and ineffective breathing, poses the challenge. It is difficult to distinguish between these infants and those with an increased work of breathing but maintaining effective respiratory effort, who may be successfully treated with CPAP. Further, the limited available data suggests that the accuracy of the clinical assessment of respiratory effort and chest wall excursion in preterm infants is poor [31,32], adding additional subjectivity to this assessment.

In the NRP algorithm, apnea, gasping, and HR <100 bpm are indications for initiating IPPV. We have defined the indications for enrolling and randomizing an infant in the SAIL trial using the same terminology. This enables inclusion of infants with obvious inadequate respiratory effort as well as infants with persistent but ineffective respiratory effort, as suggested by HR <100 bpm. Keeping the language of this initial assessment consistent with the NRP algorithm allows providers to determine eligibility for trial enrollment based on the same criteria they currently use when deciding to perform IPPV during DR resuscitation.

### Potential impact

Survivors with BPD often suffer serious pulmonary and/or neurodevelopmental sequelae, and the overall annual cost of treating BPD in the United States is $2.4 billion [33]. While extremely low birth weight (<1000 g at birth) infants are at high risk for developing BPD or death, the most vulnerable are those born between 23 and 26 weeks gestation. Despite a significant amount of research designed to prevent BPD, there has been little improvement in the incidence or severity of the disease. Sustained inflation is a promising new intervention that may reduce this burden. The SAIL trial will provide relevant and timely evidence for the efficacy and safety of sustained inflation in extremely preterm infants.

## Trial status

At the time of this submission, this trial has been approved by local Ethics Boards and is recruiting subjects at selected study sites.

## Additional files

Additional file 1: Table S1. List of Approving Ethical Committees. Additional file 2: Table S2. Secondary outcomes of the SAIL trial. Additional file 3: Table S3. Additional data collected during the study period [34]. Additional file 4: Table S4. Stopping Rules for Interim Analyses.

## Abbreviations

BPD: Bronchopulmonary dysplasia
CPAP: Continuous positive airway pressure
DR: Delivery room
DSMC: Data safety monitoring committee
FRC: Functional residual capacity
GA: Gestational age
HR: Heart rate
IPPV: Intermittent positive pressure ventilation
PEEP: Positive end expiratory pressure
PMA: Postmenstrual age
RCT: Randomized controlled trial
SI: Sustained inflation
