# Supplementary material for: Sustained Aeration of Infant Lungs (SAIL) trial: study protocol for a randomized controlled trial
Source: Trials. 2015 Mar 15;16:95. doi: 10.1186/s13063-015-0601-9 (PMC4372179; doi:10.1186/s13063-015-0601-9)
Supplement: Additional file 1: Table S1. — List of Approving Ethical Committees. [file 13063_2015_601_MOESM1_ESM.doc]

Additional file 1: Table S1: List of Approving Ethical Committees

| Institution | Date of Approval | Reference Number |
| --- | --- | --- |
| University of Pennsylvania, Philadelphia PA, USA | 6 May 2014 | Protocol Number 819176 (2 Clinical Sites) |
| University of Pennsylvania, Philadelphia PA, USA | 27 May 2014 | Protocol Number 819208 (Data Coordinating Center) |
| Woman & Infants Hospital of Rhode Island, Providence RI, USA | 13 June 2014 | Project Number 14-0052 |
| University of California- Davis, Sacramento CA, USA | 28 August 2014 | IRB ID 640318-2 |
| Children’s Hospital University of Ulm, Ulm Germany | 6 October 2014 | Application number 265/14 |
| Ospedale dei Bambani, Milan Italy | 18 October 2014 | Number of Register of opinions: 495-092014 |
| Leiden University Medical Center, Leiden the Netherlands | 29 October 2014 | Reference P14.182/NV/gk |
| Royal Alexandra Hospital, Edmonton Canada | 4 December 2014 | Pro00052888 |
| Emma Children’s Hospital, Amsterdam the Netherlands | 8 December 2014 | Reference number 2014_377#A2014102 |
| The Royal Women’s Hospital, Melbourne Australia | 7 January 2015 | Project 14/31 |
| Tufts Medical Center, Boston MA, USA | 19 February 2015 | IRB # 11456 |
| Sharp Mary Birch Hospital for Women and Newborns, San Diego CA, USA | 20 February 2015 | IRB #130791 |
