# Supplementary material for: Sustained Aeration of Infant Lungs (SAIL) trial: study protocol for a randomized controlled trial
Source: Trials. 2015 Mar 15;16:95. doi: 10.1186/s13063-015-0601-9 (PMC4372179; doi:10.1186/s13063-015-0601-9)
Supplement: Additional file 2: Table S2. — Secondary outcomes of the SAIL trial. [file 13063_2015_601_MOESM2_ESM.doc]

Additional file 2: Table S2: Secondary outcomes of SAIL trial

| Time point | Outcomes |
| --- | --- |
| Initial delivery room (DR) resuscitation | - Heart rate in the delivery room (DR) - Pressure-volume characteristics in the DR (selected sites only) - Need for intubation in the DR - Chest drain placed in the DR |
| First 10 days of life | - Need for intubation in the first 24 hours of life - Use of inotropes - Pneumothorax or new chest drains in the first 48 hours of life, duration of chest drains in situ - Pulmononary interstitial emphysema - Supplemental oxygen use: Oxygen profile over first 24 hours post-DR using hourly FiO_2_ records: Highest FiO_2_ for first 48 hours of life, Highest FiO_2_ and area under the curve FiO_2_ for first week of life - Imaging findings of intraventricular hemorrhage (especially grade 3 and 4) by 48 hours and by day 10 of life - Death or need for invasive mechanical ventilation at 7 days |
| Hospital stay | - Individual components of primary outcome - Survival to discharge without BPD, retinopathy of prematurity (stage 3 or higher or requiring treatment), or significant brain injury (IVH grade 3 or 4, periventricular leukomalacia) - Retinopathy of prematurity stage 3 or higher, or requiring treatment - Pneumothorax and pulmonary interstitial emphysema (PIE) - Duration of respiratory support (ventilation, non-invasive support, supplemental oxygen) - Use of postnatal steroids for treatment of BPD - Death before discharge - Length of hospital stay |
| 22-26 months corrected GA | - Neurodevelopmental outcome - Respiratory outcome |
