# Supplementary material for: Sustained Aeration of Infant Lungs (SAIL) trial: study protocol for a randomized controlled trial
Source: Trials. 2015 Mar 15;16:95. doi: 10.1186/s13063-015-0601-9 (PMC4372179; doi:10.1186/s13063-015-0601-9)
Supplement: Additional file 3: Table S3. — Additional data collected during the study period [34]. [file 13063_2015_601_MOESM3_ESM.doc]

Additional file 3: Table S3: Additional data collected during study period

| **Data Collection Topic** | **Specific Data Elements** | **Time of Data Element** |
| --- | --- | --- |
| **Maternal Data Form** | Maternal age, gravida parida status, exposure to antenatal steroids and type; maternal ethnicity; maternal education, any medications given to prolong pregnancy; placental abruption; chorioamnionitis; rupture of membranes > 24 hours before delivery; mode of delivery | Completed within one week of enrollment |
| **Screening and Enrollment Form** | Meets inclusion criteria and none of exclusion criteria, date and time of randomization, and treatment allocation | First 30 seconds of birth |
| **Resuscitation Intervention Form** | Exact time, duration, and pressure of sustained inflations (intervention arm), exact time and pressures of PPV (control arm) | First 2 minutes of life |
| **Delivery Room Data Form** | Date and time of birth, birth weight, sex, time of cord clamping, Apgar scores at 1 and 5 minutes, respiratory interventions performed, procedures performed, medications given, final respiratory status, and outcome of resuscitation | First hour of life |
| **SNAPPE Score (34)** | Severity score compiled from including lowest pH, PaO_2_ and corresponding FiO_2_ and MAP, urine output and presence of seizures | Data from first 12 hours of life |
| **Respiratory Status until 36 wks PMA** | Current respiratory status at once/week to include: mode of respiratory support, specific ventilatory settings, infant’s respiratory rate, highest FiO_2_ from previous time point and corresponding SpO_2_, evidence of new air leak, date of successful extubation | Daily for the first week, then weekly until 36 weeks PMA |
| **Respiratory Status at 36 wks PMA** | Exact 36 wks PMA date, respiratory support, If applicable pass/fail Oxygen Reduction Test (ORT) | Can be completed within one week of reaching 36 wks PMA |
| **Clinical Assessment** | Weight, head circumference (at birth, then weekly), medications, blood products, nutrition (type and amount of enteral and parental nutrition) | Daily for first 10 days, then weekly until 36 wks PMA |
| **Imaging Data** | Head ultrasound and brain MRI findings, abdominal films with evidence of necrotizing enterocolitis | Daily for first 10 days, then at discharge, 44 weeks PMA, or death, whichever if first |
| **Neonatal Outcome Data** | Clinical data: PDA (treated Y/N), IVH (grade), ROP stage > 1, BPD, and positive blood culture sepsis, and other complications | Discharge, 44 weeks PMA or died, whichever is first |
| **Late Outcome Data** | Final date of discharge, transfer or death if after 44 weeks PMA; reason for continued hospitalization beyond 44 weeks PMA | For infants who remain in hospital past 44 weeks PMA |
| **Discharge Status** | Clinical data: date of discharge or death, co-enrolled in other clinical trials, and respiratory status | At discharge, 44 weeks PMA or died, whichever is first |
| **Record of Death** | Clinical: date of death, primary cause of death; autopsy performed, if yes, provide narrative | If applicable |
| **Follow-up Assessment** | Ambulatory and non-ambulatory CP defined by GMFCS, hydrocephalus requiring shunt, microcephaly, or seizure disorder, history of readmission for respiratory disease, individual components of the composite outcome of NDI or death, including BSID III cognitive, language and motor scores at 2 SD cut-offs (<70) | 22-26 month corrected GA follow-up visit in clinic |
| **Adverse Events** | Including: Fi0_2_ ≥ 40% for ≥2 hours in the first 48 hours of life; presence of air leak (pneumothorax, pulmonary interstitial emphysema, pneumopericardium), death, IVH (any grade) | Specific events during the first 10 days of life, then continuing AE reporting until 36 weeks PMA |
