# Supplementary material for: Sustained Aeration of Infant Lungs (SAIL) trial: study protocol for a randomized controlled trial
Source: Trials. 2015 Mar 15;16:95. doi: 10.1186/s13063-015-0601-9 (PMC4372179; doi:10.1186/s13063-015-0601-9)
Supplement: Additional file 4: Table S4. — Stopping Rules for Interim Analyses. [file 13063_2015_601_MOESM4_ESM.doc]

Additional file 4: Table S4: Stopping Rules for Interim Analyses

| Indication | Established Rule |
| --- | --- |
| Clear Superiority | α =0.0002 for the initial interim analysis  α=0.012 for the second analysis |
| Inferiority | Power=0.8 to demonstrate a 15% point increase in the risk of outcome from 65% in the standard therapy to 80% in the intervention group. |
| Futility | Early stopping based on futility of the primary outcome will not be considered independently of the secondary clinical and safety outcomes. |
